# ΩqPCR measures telomere length from single-cells in base pair units

**DOI:** 10.1093/nar/gkab753

**Published:** 2021-09-17

**Authors:** Fusheng Xiong, Wayne D Frasch

**Affiliations:** School of Life Sciences, Arizona State University, PO Box 874501, Tempe, AZ 85287-4501, USA; School of Life Sciences, Arizona State University, PO Box 874501, Tempe, AZ 85287-4501, USA

## Abstract

ΩqPCR determines absolute telomere length in kb units from single cells. Accuracy and precision of ΩqPCR were assessed using 800 bp and 1600 bp synthetic telomeres inserted into plasmids, which were measured to be 819 ± 19.6 and 1590 ± 42.3 bp, respectively. This is the first telomere length measuring method verified in this way. The approach uses Ω-probes, a DNA strand containing sequence information that enables: (i) hybridization with the telomere via the 3′ and 5′ ends that become opposed; (ii) ligation of the hybridized probes to circularize the Ω-probes and (iii) circularized-dependent qPCR due to sequence information for a forward primer, and for a reverse primer binding site, and qPCR hydrolysis probe binding. Read through of the polymerase during qPCR occurs only in circularized Ω-probes, which quantifies their number that is directly proportional to telomere length. When used in concert with information about the cell cycle stage from a single-copy gene, and ploidy, the MTL of single cells measured by ΩqPCR was consistent with that obtained from large sample sizes by TRF.

## INTRODUCTION

The ends of linear chromosomes are stabilized by telomeres comprised of very long stretches of short repeat sequences of DNA, which is TTAGGG in humans (1). Somatic cell replication progressively shortens telomeres until telomere length becomes critically short, at which point cells enter replicative senescence that leads to cell death (2,3). Normal telomere shortening limits cellular life span and prevents tumorigenesis. However, aberrantly short telomere length is implicated in aging-related diseases such as cancer (4–6), as well as cardiovascular disease (7–9), and is associated with increased mortality (10–12). Conversely, in stem cells that require renewal, telomere length is maintained by telomerase, a ribonucleoprotein containing the RNA template *TERC*, and telomere reverse transcriptase (TERT) to delay senescence (13,14). Telomerase is activated in >90% of human tumors (4), which promotes cell proliferation and survival (12,13), and some forms of cancer are believed to result from excessively long telomeres due to excessive expression of TERT (15,16).

In the widely used TRF Southern blot technique to measure telomere length (17,18), restriction fragments are separated by electrophoresis, hybridized to radiolabeled probes, then visualized by autoradiography. A densitometry scan of the radiogram provides a distribution of the lengths of telomeres in the sample, which is used to estimate mean telomere length. TRF requires 0.5–5 μg of DNA, which is equivalent to the DNA from ∼76 000–760 000 cells with diploid genomes. The procedure takes several days to complete, which limits the number of samples that can be analyzed per week by a skilled technician to ∼130 (19). Relative telomere length can be determined from single cells that are in metaphase using Q-FISH (4,20,21). However, this time intensive method is limited to actively dividing cells, which eliminates the ability to measure telomere length of many types of post-mitotic, differentiated and senescent cells among others. Modified FISH methods (4,20,21) can measure telomere lengths in non-dividing and senescent cells, but require large cell populations. Unfortunately, FISH approaches can have > 2–10 kb measurement variations, which can exceed the lengths of telomere in human cells (21). The time-intensive nature of these approaches also severely limits the scalability of sample analysis. Since a clinical tissue sample may contain a mixture of malignant and healthy cells, a single-cell approach is highly preferable to identify the cells with aberrantly long or short telomeres.

A scalable qPCR-based method known as MMqPCR has been developed that provides relative telomere length measurements in terms of a T/S ratio derived from the threshold quantification cycle (C_q_) values of the telomere primers (T) versus that of a reference gene (S) (22,23). This approach has been documented to have reproducibility problems that result from a wide variety of conditions (24–27). Although MMqPCR requires less tissue than TRF, the minimum of 35 ng of DNA is still equivalent to ∼11 000 diploid cells. Relative telomere lengths from single cells have been possible using MMqPCR, but only after both the target, and a multi-copy reference gene have been amplified by several rounds of PCR (28). Unfortunately, the repeating nature of the short telomeric DNA sequence (TTAGGG)_n_ enables telomere-specific PCR primers to hybridize in a plethora of combinations staggered along the length of the telomere. As a result, heterogeneous amplification reactions occur simultaneously that decrease the precision and accuracy of the subsequent telomere measurement.

We now report the scalable method of ΩqPCR to determine absolute telomere length from single-cells in a way that does not require PCR amplification of the sample prior to analysis. The approach uses the Ω-probe, an omega-shaped DNA strand containing sequence information that enables it to hybridize to telomere sequences in a manner that forces the 3′ and 5′ ends to be opposed. Hybridization-dependent ligation of these ends circularizes the probe, which only then can undergo PCR amplification with qPCR reagents. The number of circularized probes formed, as determined by qPCR, provides a direct measure of telomere length in units of base pair. We verified the ability of ΩqPCR to measure telomere length accurately and precisely using synthetic telomere sequences of known length.

## MATERIALS AND METHODS

### Cell culture

The human cell lines used for telomere length measurements were the result of a generous gift from Dr Laimonas Kelbauskas at Arizona State University who obtained them from the American Type Culture Collection (ATCC, Rockville, MD, USA). All cells lines used were newly purchased, and authentication of the cell line was conducted by the ATCC.

### Single cell isolation by stochastic seeding at limiting dilutions

Single human cells were isolated using the Terasaki plate mediated protocol (29,30). Terasaki-style microtest plates were briefly cleaned using pressurized nitrogen gas to remove particulate from the well area. The outer surfaces of the plates were sprayed with 70% ethanol and allowed to dry in a sterile, laminar flow hood prior to cell seeding. The cultured cells were trypsinized with 0.05% trypsin–EDTA for 10 min, centrifuged at 900 rpm for 3 min, and counted using the Trypan Blue assay on a Countess^®^ automated cell counter (Life Technologies, Carlsbad, CA, USA). Cells were resuspended at 200–300 cells/ml, which is the optimal cell density required to achieve one single cell per well in a Terasaki plate, and 10 μl of the thoroughly-homogenized suspension was dispensed into each well. After incubation for 20 min in a 37°C incubator, the Terasaki plate was observed by phase contrast microscopy on a Nikon TS-100 microscope with 10× and 20× objectives to verify occupancy of no more than a single cell in each well, and scored for viability as ‘live’ or ‘dead’ based on spreading morphology and phase contrast characteristics. In the resultant well occupancy, 15–25 of the wells per plate contained a single cell, which was a distribution expected by Poisson statistics with high reliability (*R*^2 ^> 0.98).

### Single cell lysis

Each sorted single-cell together with 10 μl of PBS was manually removed from its well with a 190 μm diameter capillary tip (Drummond Scientific), and transferred into a nonstick 0.25 ml PCR tube (Axygen Scientific). After centrifugation, and removal of the supernatant, the cell was placed in 3 μl of a solution of 200 mM KOH and 50 mM dithiothreitol, centrifuged, and heated at 65°C for 10 min. Subsequently, 3 μl of neutralization buffer (300 mM KCl, 200 mM HCl, and 900 mM Tris–HCl, pH 8.3) was added. Samples were then analyzed by ΩqPCR, or if not analyzed immediately, were stored at −20°C until the ΩqPCR assays were performed.

### Hybridization and ligation of Ω-probes

To hybridize Ω-probes to telomere repeat sequences of genomic DNA from each lysed cell, the lysate was first denatured at 95°C for 2.5 min, and quickly put on ice to prevent natural annealing. The denatured cell lysate was warmed to 55°C, and 1 μl of 100 nM Ω-probes that had been pre-equilibrated at 55°C were added to the cell lysate, mixed, and incubated for 30 min. Hybridization occurred under a programmed ramping step of 1°C/1.5 min from 55°C to 16°C. Hybridized Ω-probes were circularized by ligation at 16°C for 5 min after addition of 1 μl of 10× Quick Ligation Buffer (Thermo Scientific), 1 μl of 400 kU/ml of T4 DNA ligase, 0.5 μl of 250 μM ATP, and 0.5 μl of 1 M dithiothreitol.

### Single-cell qPCR assays

For ΩqPCR assays, 10 μl of Thermo Scientific qPCR reaction mixture (2X PCR Master Mix, 250 nM *Taq*Man^®^ 6FAM dye hydrolysis probe, as well as 500 nM each of the Pf and the Pr primers (Table 1) was added to each sample containing circularized Ω-probes. The 36B4 gene located on Chromosome 12 that encodes a 60S human acidic ribosomal phosphoprotein PO was amplified using the SCG-Pf and SCG-Pr primers (Table 1) for quantification of the pg of gDNA in single cells. The conditions of qPCR amplification of 36B4 were the same as described for the ΩqPCR assays.

**Table 1.** Oligonucleotide sequences used in the ΩqPCR assay

Oligonucleotide	Sequence
Ω-Probe	*Pi*AACCCTAACCCTAACCCCGCGCTAGACTAAGCGCTCCAGTGACTCAGCAGCTACCCGGCAACTAGATGCCGCCCCTAACCCTAACCCT
Ω-Pf^a^	CAGTGACTCAGCAGCTACCCG
Ω-Pr^b^	GAGCGCTTAGTCTAGCGCG
SCG^c^-Pf	CAGCAAGTGGGAAGGTGTAATCC
SCG^c^-Pr	CCCATTCTATCATCAACGGGTACAA
Hydrolysis probe^d^	6FAM-CAACTAGATGCCGCCC-MGBNFQ
Ω-ORF	CAGTGACTCAGCAGCTACCCGGCAACTAGATGCCGCCCCTAACC
	CTAACCCTAACCCTAACCCTAACCCCGCGCTAGACTAAGCGCTC

^a^forward primer sequence; ^b^reverse primer binding sequence; ^c^single-copy gene 364B; ^d^*Taq*Man

Amplification of circularized Ω-probes by ΩqPCR was carried out in a 96-well Falcon microtiter plate using an ABI 7500 Fast Real-Time PCR system (PE Applied Biosystems). The two-step thermal cycling consisted of an initial 20 s denaturation at 95°C followed by 45 cycles of 95°C for 3 s, and 58°C for 30 s with fluorescence acquisition during the 58°C amplification step. PCR amplifications were detected directly by monitoring the increase in fluorescence from the dye-labeled hydrolysis probe (*Taq*Man) reporter which is an ΩqPCR probe-specific oligonucleotide with a 5′-reporter dye (FAM-6-carbooxy-fluorescein), and a 3′-quencher dye (TAAMRA-6-carboxy-*N*,*N*,*N*,*N*-tetramethylrhodamine).

The Ω-probes and Ω-open reading frames (Ω-ORFs), were obtained by custom DNA synthesis. These DNA sequences arrived as freeze-dried samples in vials that specified the total amount in nmol. A stock solution of each was prepared by dissolving the entire content of each vial. The standard curves of Figure 4 were derived from serial dilution of the Ω-ORF stock solution.

### Validation of ΩqPCR

To validate the accuracy and precision of ΩqPCR to measure telomere length, 0.125 pg and 0.25 pg of DNA from the pSXneo135 or pSXneo270 plasmids, respectively, were incubated with 1 μl of 100 nM Ω-probes following the same hybridization, circularization, and ΩqPCR protocols applied for the single-cell samples. The pSXneo135, and pSX270 plasmids contain 800 and 1600 bp long telomere sequences, respectively. These plasmids were generated from the pSP 74 (31), which contains 2400 bp, such that 1 μg of the pSXneo135 and pSXneo270 contain 2.86 × 10^11^ and 2.29 × 10^11^ molecules of DNA, respectively.

### TRF assay

Telomeric restriction fragment (TRF) length assays were performed using the *TeloTAGGG* Telomere Length Assay Kit (Roche) as described in the protocol provided by the manufacturer. In brief, 2.5 μg of genomic DNA was digested at 37°C for 6 hours with mixing *Hinf* I (20 MU) and *Rsa* I (20 MU), and the digested DNA fragments were separated by electrophoresis on a 0.8% agarose gel in 1× TAE buffer (pH 8.0). After electrophoresis, the DNA in the gel was depurinated in 0.25 N HCl for 10 min, denatured in 0.5 N NaOH, 1.5 M NaCl for 2 × 15 min, and then transferred to immobilon-NY^+^ membrane (Millipore-Sigma-Aldrich, Ref. No. 11209299001) using 20× SSC transfer buffer. After transfer, the DNA was fixed on the membrane by UV-crosslinking for 10 m, pre-hybridized with DIG Easy Hyb at 42°C for 1 h with gentle agitation, and then hybridized with telomere-specific DIG-labeled probe (1μl per 5 ml fresh DIG Easy Hyb) under the same conditions for 3 h. The hybridized DNA was washed twice with a buffer containing 0.3 M NaCl, 30 mM sodium citrate, and 0.1% SDS at room temperature for 15 min and then twice with a buffer containing 30 mM NaCl, 3 mM sodium citrate, and 0.1% SDS at 50°C for 15 min. After 30 m blocking in 50 ml freshly prepared 1× blocking solution, the membrane was incubated with anti-DIG alkaline phosphatase (1:10 000) at room temperature for 30 min. After washing twice for 15 min, the membrane was incubated with CDP-Star at room temperature for 5 min. The telomere signals were detected via chemiluminescence scanning using a ChemiDoc Imaging System (Azure 600). Image quantification was performed using ImageJ (NIH) software to calculate the average telomere lengths (32). Measurement of telomere length by the Southern blot analysis of terminal restriction fragment lengths were carried out as previously described (33).

## RESULTS

### Design and mechanism of ΩqPCR telomere length measurements

The ΩqPCR assay enables the computation of telomere length in units of base pair as the result of three simple steps. First, Ω-probes are hybridized to the telomere sequences of genomic DNA under conditions in which the probes cover the entire length of the telomeres. Second, only the Ω-probes hybridized to the telomeres can be ligated to circularize them. Third, only the circularized probes are capable of qPCR, which quantitates the number of Ω-probes that had hybridized to the telomere. Since each Ω-probe occludes a specific number of bases along the telomere, computation of the length of the telomere is a function of the number of circularized probes.

**Figure 1.**
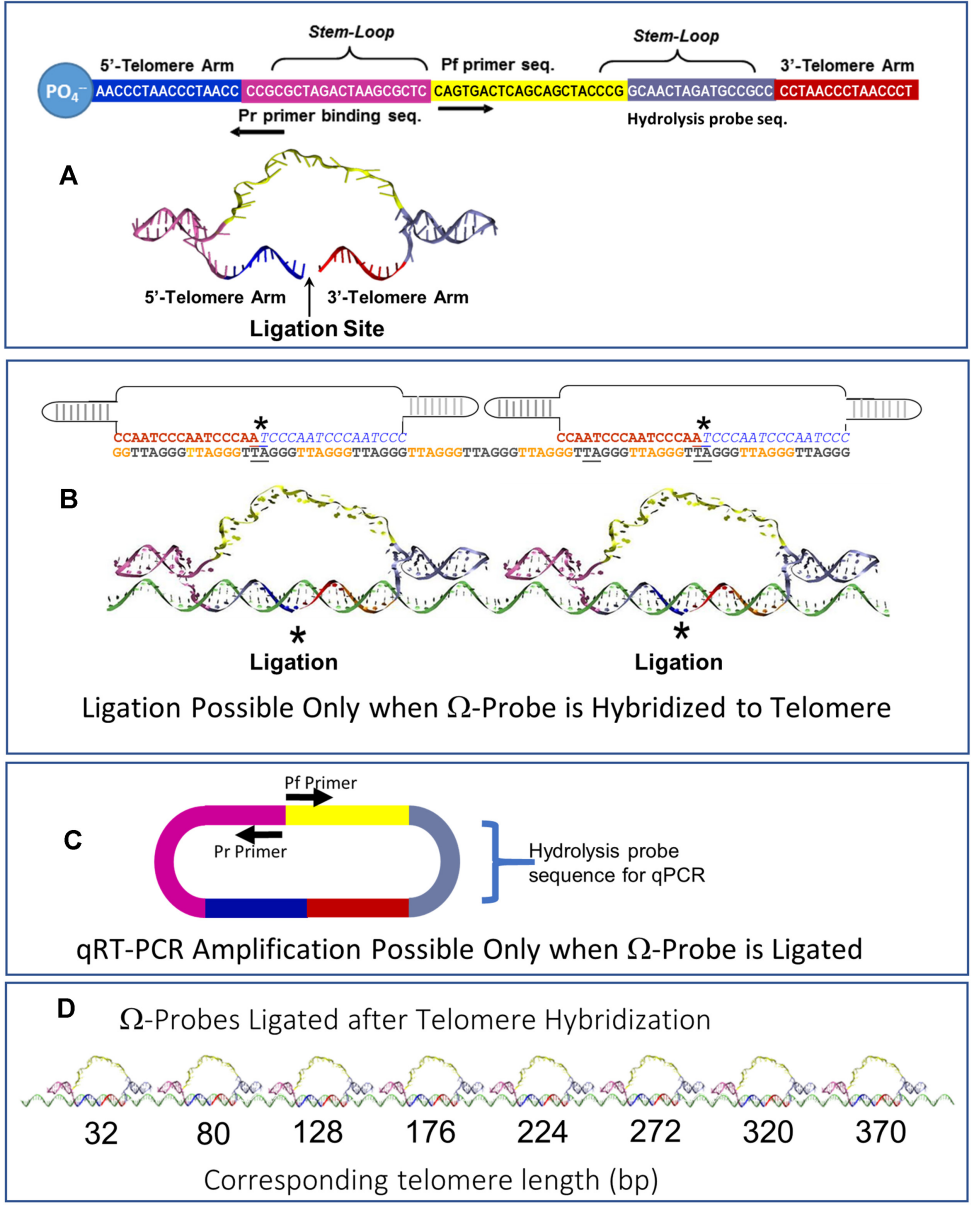
(**A**) Unfolded and folded forms of the Ω-probe showing the color-coded sequences of: arms that hybridize the telomere; stem–loops; hydrolysis probe sequence; and the qPCR forward and the reverse primer-binding sequences. (**B**) Two Ω-probes hybridized to adjacent position on the telomere sequence as a molecular model or as a diagram where (*) indicates ligation sites. (**C**) Ω-probes can only be amplified by qPCR after hybridization-dependent circularization. (**D**) Telomere length is calculated using ΩqPCR by quantifying the number of circularized Ω-probes.

Several design features incorporated into the sequence of the Ω-probe enable the accurate computation of telomere length (Figure 1A). The stem-loops force the 16-base long 3′ and 5′ arms of the Ω-probe to hybridize to a telomeric DNA strand, such that the Ω-probe 3′-end and phosphorylated 5′-end face each other in a conformation that enables the ligase-dependent circularization of each hybridized Ω-probe. For successful ligation, the hybridized bases must be complementary at the ligation site. For this reason, the ligation site was positioned complementary to the telomere TA base sequence (GGGTTAGGG) to minimize ligation of Ω-probes hybridized to the subtelomeric stretches that have the (TTXAGGG)_*n*_ repeat sequence (Figure 1B). Each Ω-probe contains a forward primer sequence, and a reverse primer binding sequence necessary for qPCR, as well as the hydrolysis probe binding sequence. Since Ω-probe ligation-dependent circularization is required for the reverse primer to function during qPCR (Figure 1C), only the Ω-probes that were both correctly hybridized to the telomere, and circularized can be amplified upon addition of primers and hydrolysis probes.

To ensure full coverage of the entire length of the telomeres by Ω-probes, hybridization was allowed to occur slowly in the presence of a saturating amount of the probes. Each circularized probe hybridizes to a total of 32-bases (Figure 1D), and their adjacent stem-loops occlude the telomere sequence by an additional 16-bases (7 bases per stem-loop) where two Ω-probes abut on the telomere. In this manner telomere length per copy of genomic DNA is calculated in units of base pair from the number of circularized Ω-probes quantified by qRT-PCR (ΩqPCR) using Equation (1), where TL is telomere length, and N_cp_ is the number of circularized Ω-probes.

(1) $$\begin{equation*}{\rm{TL}} = 48\left( {{{\rm{N}}_{{\rm{cp}}}}-1} \right) + 32\end{equation*}$$

### ΩqPCR telomere measurement validation using synthetic telomeres of known length

**Figure 2.**
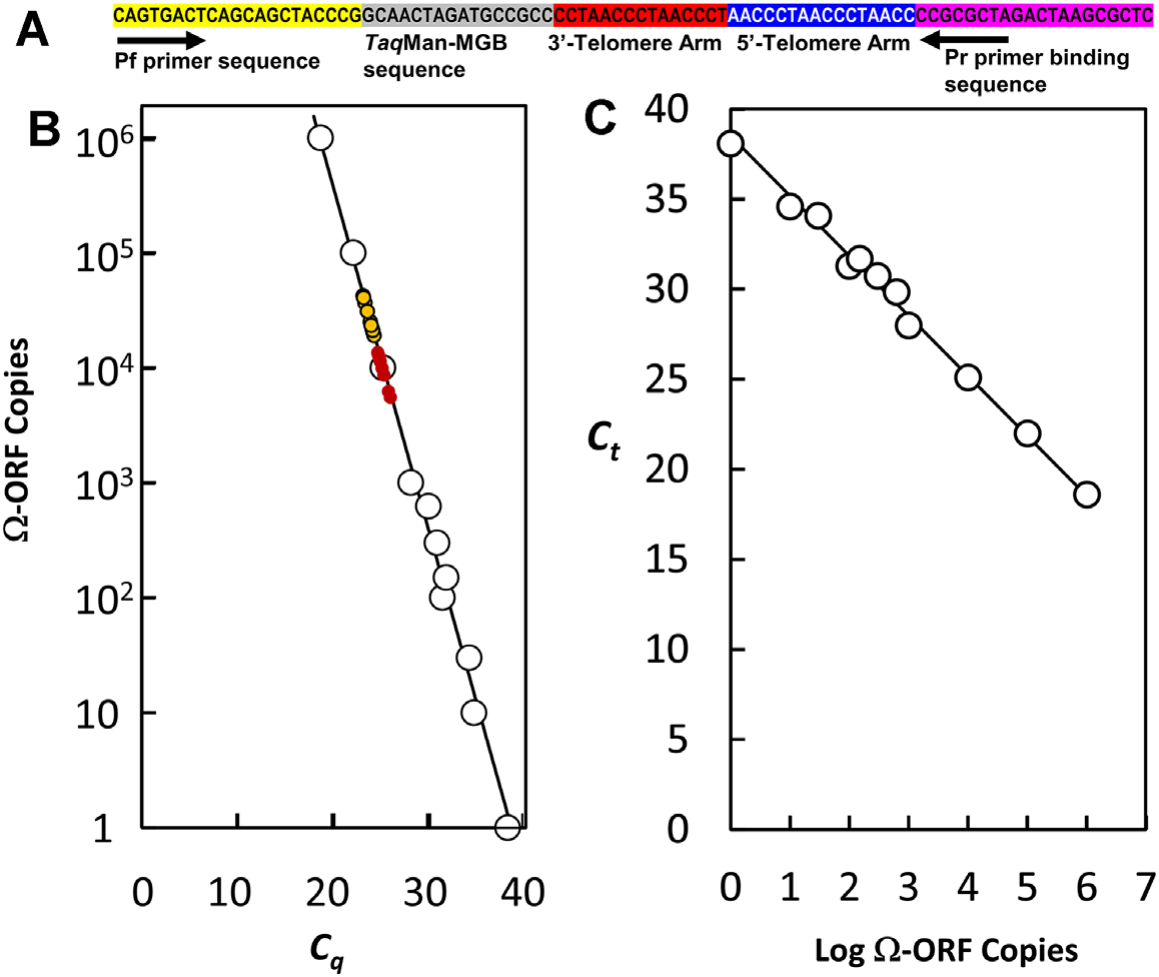
(**A**) Sequence components of the Ω-open reading frame (Ω-ORF). The components are the same as those in Figure 1A, but the order was rearranged so that forward primer (*Pf*) and reverse primer binding sequences (*Pr*) are at the ends to enable the Ω-ORF to undergo qPCR amplification comparable to that of the circularized Ω-probe. (**B**) qPCR standard curve of copies of the Ω-ORF versus *C_q_* where each data point (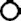) was the average of three separate measurements for which standard deviations were smaller than the size of the symbols. Distributions of *Cq* values from Figure 5B for MCF-7 (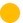), and MDA-MB-231 (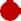) cell lines determine number of circularized ΩΩ-probes generated. (**C**) Efficiency plot for ΩqPCR based on the data of (B). Based on the derived slope of –3.3649, the calculated efficiency was 98.2%.

The relationship between the number of circularized Ω-probes, and the *C_q_* values obtained from ΩqPCR was determined using the Ω-Open Reading Frame (Ω-ORF, Figure 2A) to generate a standard curve (Figure 2B). The Ω-ORF contains the same sequence components as the Ω-probe except that the forward primer (*Pf*) and the reverse primer binding (*Pr*) sequences are at each end. Consequently, the Ω-ORF can be amplified by qPCR comparable to that of the circularized Ω-probes. The qPCR efficiency was 98.2% as calculated from the log of Ω-ORF copies versus *C*_q_ values (Figure 2C). The dependence of *C_q_* values *versus* the log of the Ω-ORF copy number (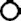) was linear over a range of six orders of magnitude with an *R*^2^ = 0.99. The number of circularized Ω-probes was calculated from the standard curve of Figure 2B that fit to Equation (2):

(2) $$\begin{equation*} {\rm N_{\rm CP}} = (4 \times {10^{11}}){\rm{ex}}{\rm{p}}^{( - 0.691{\boldsymbol{Cq})}} \end{equation*}$$

**Figure 3.**
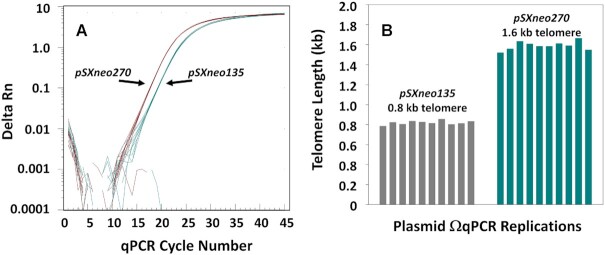
(**A**) Replications of ΩqPCR production *versus* cycle number of the 800 and 1600 bp long synthetic telomere sequences in the *pSXneo135* and *pSXneo270* plasmids. (**B**) Telomere lengths measured using ΩqPCR from each of the replications from (A) for the synthetic telomeres in the *pSXneo135* and *pSXneo270* plasmids.

Figure 3 shows the results of using ΩqPCR to calculate the length of synthetic telomere sequences known to be 800 and 1600 bp long that had been inserted into plasmids pSXneo135 and pSXneo270. The average lengths of these sequences, each measured from 10 replications by ΩqPCR, were 819 ± 19.6 (s.d.) bp and 1590 ± 42.3 (s.d.) bp, respectively. A sample calculation of the telomere length based on Equations (1) and (2) using these plasmids is shown in Table 2. The calculated lengths correspond to an average circularization of 16.7 and 33.3 hybridized Ω-probes per plasmid to the short and long synthetic telomere sequences. The standard deviation for the 1600 bp sequence corresponds to telomere length occluded by one Ω-probe (48 bp), while that of the 800 bp sequence is equivalent to that of one stem loop of one Ω-probe (16 bp). To our knowledge, this is the only telomere length measurement technology where the accuracy and precision have been verified against synthetic telomere sequences of known length.

**Table 2.** Calculations to derive telomere length of synthetic telomeres by ΩqPCR

Plasmid		pSXneo135	pSXneo270
Known telomere length		800	1600
pg of Plasmid DNA per sample		0.125	0.250
Plasmid molecules per sample		35750	57250
*C_q_*		19.380	17.741
No. circularized Ω-probes	*N*_CP_ = (4 × 10^11^)exp^(–0.691^*^Cq^*^)^	610 338	1 896 839
Total telomere length (bp)	TL = 48(N_CP_ − 1) + 32	2 926 214	91 048 257
Per plasmid telomere length (bp)	PPTL = TL/plasmid copies	819	1590

### ΩqPCR telomere length measurements from single cells

**Figure 4.**
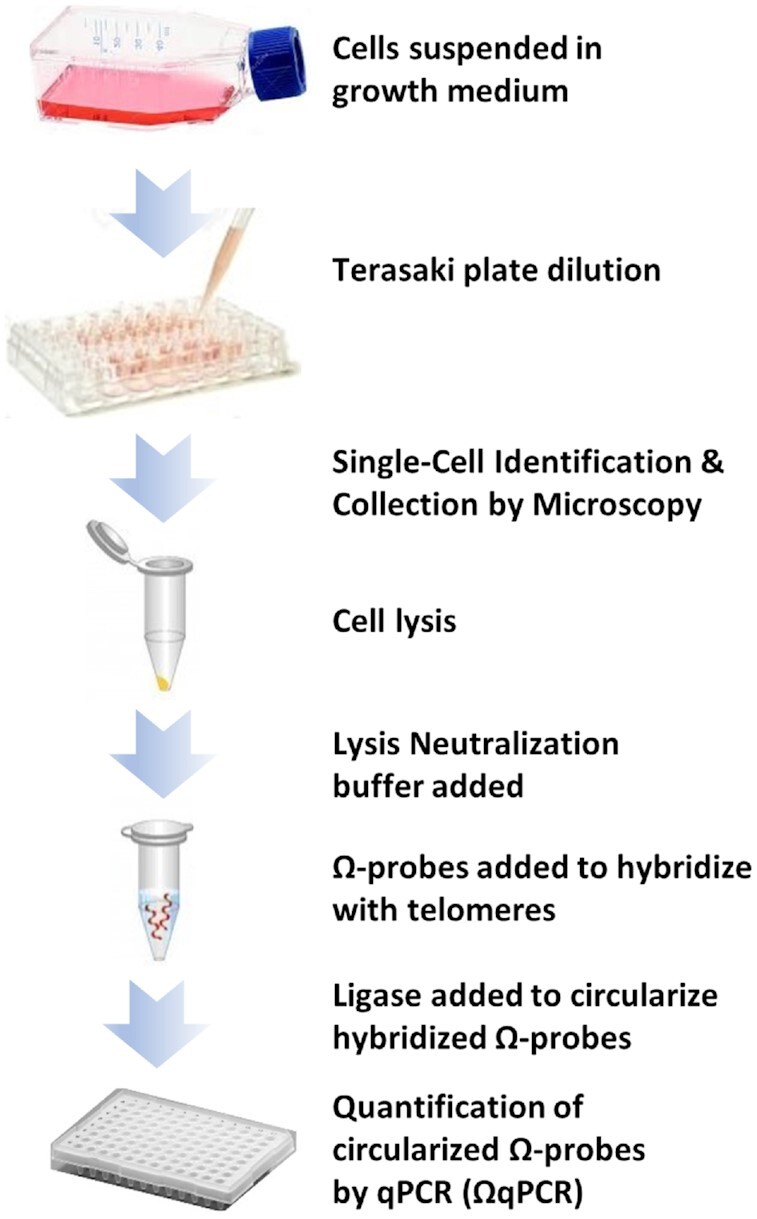
Workflow for analysis of telomere length using ΩqPCR from the genome of a single cell.

Ten single cells from each of five different human cell lines were sorted by the Terasaki Plate-mediated protocol (30) into 96-well Falcon microtiter dishes where the cells were allowed to adhere to the inner surface of the wells (Figure 4). Wells that contained a single cell were verified by microscopy. Each cell was centrifuged, growth medium removed, and lysis buffer was added, which was subsequently neutralized. The time required to remove the cells from the vial supplied by the ATCC until the addition of lysis buffer was ∼20 min.

**Figure 5.**
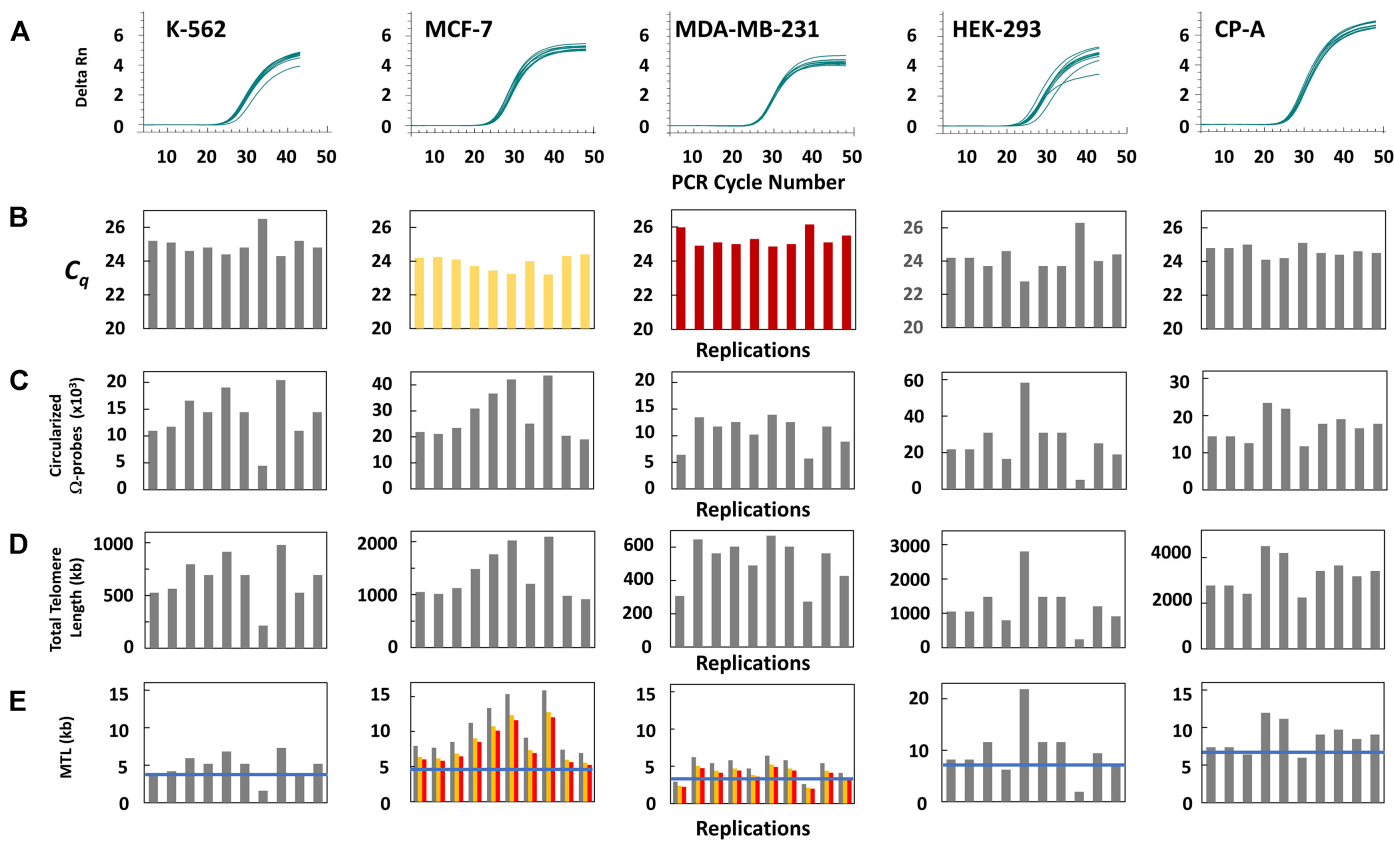
(**A**) Ten replications of single-cell ΩqPCR shown as PCR productions versus cycle number for each of five cell lines. (**B**) Distribution of *C_q_* values derived from the data of (A) for each cell line examined. The *C_q_* values of negative controls ranged from 40–42. (**C**) Distribution of the number of circularized Ω-probes from single-cell measurements calculated from (B) using Equation (2). (**D**) Distribution of total telomere lengths calculated from (C) using Equation (1). (**E**) Distribution of mean telomere lengths (MTL) calculated from (C) based on karyotype information for each cell line as described in text. For cell lines MCF-7 and MDA-MB-231 cell lines, the modal (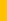), as well as the low (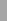), and high (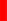) range of ploidy values were used to calculate MTLs from ΩqPCR measurements. Mean telomere lengths 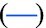
calculated from TRF data (Figure 7).

Figure 5A shows the qPCR amplification plots of the Ω-probes that had become circularized as the result of hybridization to the telomeres of the genomic DNA from each single cell. The fluorescent signals from the circularized probes in all samples were sufficient to generate qPCR amplification plots from which the *C_q_* could be determined with precision (Figure 5B). Values of C_q_ for negative controls were between 40 and 42. The number of circularized Ω-probes determined from the *C_q_* of each ΩqPCR measurement from single cells using Equation (2) is shown in Figure 5C.

Examples of the dependence of the number of circularized Ω-probes generated versus *C_q_* is shown in Figure 2B for each of the 10 cells examined from the MCF-7 (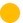), and MDA-MB-231 (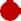) cell lines. For these cell lines, the average number of circularized Ω-probes was 23021 ± 2437 (s.d.) and 8213 ± 737 (s.d.). Of the 50 measurements made from the five cell lines, the smallest and largest numbers of circularized Ω-probes made were 4461 probes and 58,323 probes from K-562 and HEK-293, respectively. The total length of the sum of telomere regions in each cell (Figure 5D) was calculated directly from the number of circularized Ω-probes using Equation (1). In light of the dependence of the number of circularized Ω-probes generated versus *C_q_* (Figure 2B), it is clear that the measurements have enough sensitivity to distinguish small differences in telomere length, as well as the dynamic range to accommodate a wide range of telomere lengths.

**Figure 6.**
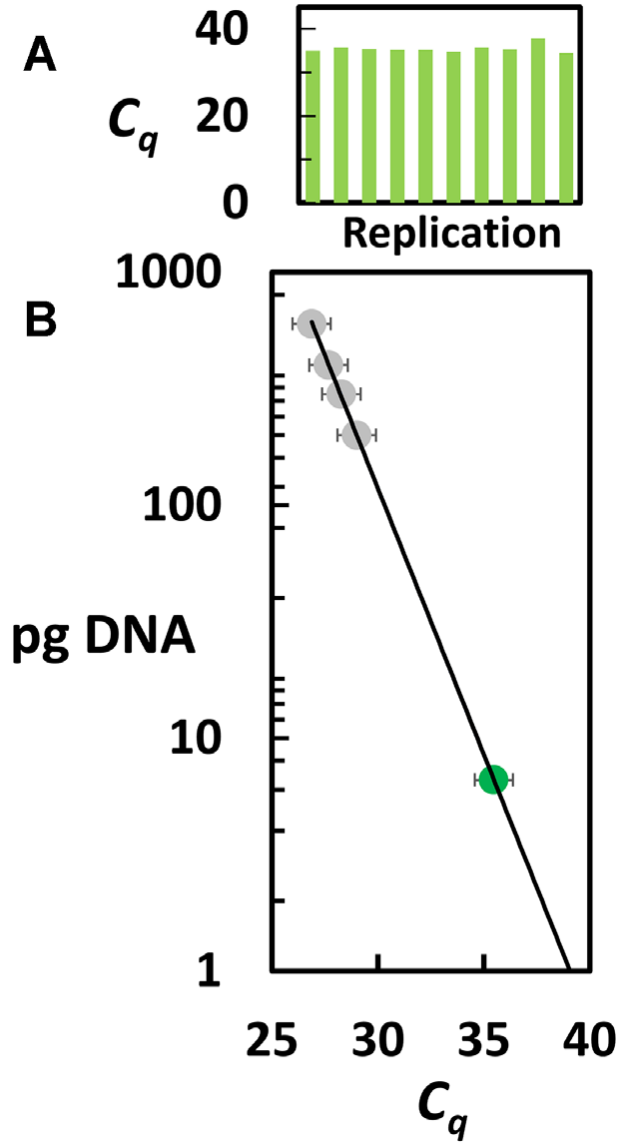
(**A**) Ten replications of single-cell qPCR of the 36B4 single-copy reference gene in CP-A cells. (**B**) qPCR standard curve of pg DNA versus *C_q_* from three replications of the 36B4 gene in purified human DNA (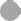). Amount of DNA in single CP-A cells (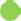) determined from the average *C_q_* value from (B). Brackets indicate s.d.

Determining the MTL from a ΩqPCR measurement requires additional knowledge of cell ploidy and cell cycle position (G1 versus G2/M). The karyotype of CP-A cells is nearly diploid, except that N20 is trisomic. Human diploid cells have been determined to contain ∼6.6 pg of DNA per cell (34). The amount of DNA in each of 10 single CP-A cells was determined by qPCR of the 36B4 single-copy reference gene located on chromosome N12 (Figure 6A). The average *C_q_* value of 35.5 ± 0.9 from the 10 replications of the 36B4 gene (Figure 6A) corresponded to the presence of 6.6 pg of DNA per CP-A cell (Figure 6B), which is consistent that found in human diploid cells in the G1 stage of the cell cycle prior to DNA replication (34). Based on these data, the CP-A cells examined contain 94 telomeres, which was used to determine mean telomere length (MTL) by ΩqPCR from each of the 10 single CP-A cells (Figure 5E).

Calculation of average telomere length of single cells from the other cell lines (Figure 5E) was based on the following karyotype information assuming that the cells were in the G1 phase. HEK-293 cells have a well-defined modal number of 64 (128 telomeres). Aneuploid MDA-MB-231 cells are near triploid cells with a modal number of 64, and a range from 52 to 68 chromosomes. The MTL for each cell examined by ΩqPCR was calculated using 104 (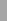), 128 (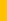), and 136 (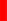) telomeres. The difference between the modal value and the high range was smaller than between the modal value and the low range. The MCF-7 cell line is also aneuploid with a modal number of 82, and a range from 66 to 87. The distribution of MTLs determined by ΩqPCR based on 132 (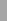), 164 (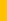), and 174 (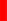) telomeres (Figure 5E) also showed a smaller difference between the modal value and the high range. Although K-562 cells are aneuploid, the modal chromosome number of 67 has been shown to be stable (35,36).

**Figure 7.**
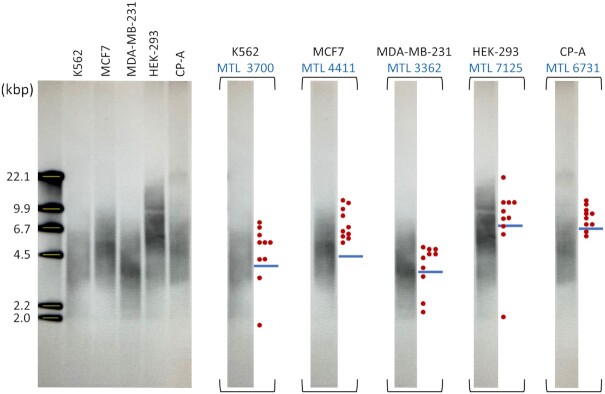
Distribution of telomere lengths determined by telomeric restriction fragment (TRF) analysis. Molecular length markers used to make the standard curve were designated by the supplier to have molecular lengths in kbp of 2.02, 2.32, 4.36, 6.66, 9.42 and 23.13. The lengths indicated at positions 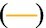
are the best fit to the standard curve. The MTLs calculated from single-cell ΩqPCR measurements (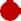) from Figure 5E are shown adjacent to the copy of each lane of the corresponding cell line for comparison along with the MTL determined by TRF 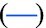.

Comparison of the distribution of telomere lengths obtained by ΩqPCR with the MTL obtained by TRF analysis (Figure 7) is shown for each cell line in Figure 5E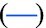. The MTL values determined by TRF for the five cell lines was 3700 (K562), 4411 (MCF7), 3362 (MDA-MB-231), 7125 (HEK-293) and 6731 (CP-A), which are shown in both Figures 5E and 7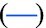 to compare directly to the distribution of telomere lengths derived by ΩqPCR. In Figure 7, the Southern blot is shown unaltered to serve as a means to construct the standard curve from the molecular length markers from which the MTL for each cell line was determined. Molecular length markers used to make the standard curve were designated by the supplier to have molecular lengths in kbp of 2.02, 2.32, 4.36, 6.66, 9.42 and 23.13. The molecular lengths indicated in Figure 7 at positions 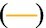 are the best fit to the standard curve, which are shown in order to make an accurate comparison to the positions of the telomere lengths calculated by ΩqPCR.

The density of DNA was scanned in each lane of the TRF blot, which was used to calculate the mean telomere length (MTL) by computational analysis that accounted for the hybridization of more probe to longer telomeres (33). The lanes of the blot for each cell line are also shown separately to enable direct comparison with mean telomere lengths measured by ΩqPCR (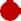) that were placed using the best fit of the standard curve to the molecular length markers. For all five cell lines, the distributions of telomere lengths calculated from the ten single cells by ΩqPCR fit within the distribution of telomere lengths measured by TRF. For MDA-MB-231 cells, the difference in MTLs determined from the modal and the high number of chromosomes varied little, and were within 10% of the MTL of 3362 bp derived by TRF (Figure 7). The MCF-7 cell line also showed a smaller difference between the modal value and the high range, with the latter, based on 174 telomeres, having the best fit to the MTL of 4411 derived by TRF (Figure 7).

## DISCUSSION

The results presented here demonstrate that ΩqPCR measures total telomere length in units of base-pair from the genomic DNA of a single cell. To our knowledge, ΩqPCR is the first method to have its efficacy verified by measuring synthetic telomeres of known length accurately and precisely. Based on the standard deviations calculated from the synthetic telomere measurements, we conclude that the variations observed in total telomere length among each set of 10 single cells from the five human cell lines examined here represent real differences between cells of the same cell line. The single-cell capability of ΩqPCR is significant because tissue samples from cancer patients contain a mixture of healthy and malignant cells that may differ significantly in telomere length. The ability to calculate telomere length from individual cells can clearly reveal differences between healthy and malignant cells rather than returning an average telomere length for the tissue sample as a whole.

Presenting the results of ΩqPCR as the total length of the sum of the telomeres per cell (Figure 5D) does not make tacit assumptions concerning the number of telomeres present. Additional information concerning the stage of the cell cycle (G1 versus G2/M), and cell ploidy is required to determine the MTL by ΩqPCR. The use of a single-copy reference gene such as the 36B4 gene used here (Figure 6) provides important information regarding the cell cycle stage, which is vital in determining the number of telomeres per cell. Since a single-copy gene measurement reports the number of copies of only the chromosome on which it resides (Chromosome 12 for 36B4), the use of karyotype information also provides important information concerning the number of telomeres. However, karyotype information is insufficient where the chromosome number is unstable. For this reason, we showed the effects of the low, modal, and high ploidy range on the MTLs calculated by ΩqPCR for cell lines MCF-7 and MDA-MB-231. Although K-562 cells are also aneuploid, the modal chromosome number of 67 has been shown to be stable (35,36). We are currently working to develop a qPCR assay capable of measuring the number of telomeres in a sample.

The MTLs of the 10 single cells calculated by ΩqPCR all correlated to the distributions of DNA density on the TRF blot for each cell line (Figure 7). In addition, in all cases, the MTL calculated from the TRF blot fit well with the lengths calculated by ΩqPCR (Figure 5E). Each lane of the TRF blot contained the DNA from ∼380,000 cells used per cell line, and revealed that telomere lengths can vary widely within each cell line. Due to the large number of cells required to observe telomere lengths by TRF, and the wide distribution of telomere lengths in a given cell line, analysis of much larger data sets by ΩqPCR is in progress to provide a better statistical comparison of the two techniques, which is beyond the scope of the current study.

The MMqPCR assay (22,23) that reports telomere length as a *C_q_* ratio relative to a single-copy gene, was modified for single-cell measurements (28). However, since this method requires ∼200-fold more genomic DNA than is present in a single human cell to give a measurable signal (34), sample DNA must first be ‘preamplified’ by several PCR cycles. Unfortunately, the entire lengths of telomeres are not amplified with fidelity due to the ability of the PCR primers to bind at many locations along the long stretches of short TTAGGG repeats. In principal, MMqPCR should be able to make single-cell measurements without preamplification because it computes telomere length from the number of annealing primers that each hybridize to ∼32 adjacent telomere bases (hybridized Ω-probes occlude 48 adjacent bases). While ΩqPCR amplifies hybridization-dependent circularized Ω-probes, MMqPCR amplifies hybridization-dependent annealing primers that can only be amplified by PCR after extension by polymerase. A possible explanation for the inability of MMqPCR to measure telomere lengths from single cells is that the *Taq* DNA polymerase that extends the primers (22) also has strong polymerization-enhanced 5′-3′ nuclease activity that concurrently cleaves and displaces each primer bound at adjacent downstream positions as the polymerase moves along the telomere template to catalyze the 5′-3′ polymerization of each upstream annealing primer. Based on the large amount of DNA required for the qPCR step, most downstream primers do not survive the polymerase/nuclease. Variability in the extent of displacement and cleavage likely compromises the accuracy and reproducibility of MMqPCR.

A similar approach to ΩqPCR was used to increase the speed and accuracy of answer determination in DNA computing to solve traveling salesman optimization problems (37). This molecular computing approach calculates the optimal answer to a given mathematical problem by assembling short DNA strands with unique sequences (comparable to the telomere repeat sequence) into long answer strands where the optimal answer is formed in greatest abundance. When optimal answers were determined by the relative intensity of bands after separation by electrophoresis (comparable to TRF analysis), answer determination required several days (38). However, the speed and accuracy were increased dramatically through the use of probes that measured abundance of the adjacent short sequences by circularization-dependent qPCR (38) of a progenitor to the Ω-probe (39).

The ΩqPCR method presented here is not limited to single-cell measurements, but can be scaled up to measure telomere length in larger tissue samples by using a single-copy gene to determine the number of cells in the sample as in Figure 6. As such, ΩqPCR provides a simple method able to calculate telomere length from all cell types accurately in units of bp, and in a timely manner that is scalable for high-sample input. Since a clinical tissue sample may contain a mixture of malignant and healthy cells, a single-cell approach is highly preferable to identify the cells with aberrantly long or short telomeres. None of the currently available assays that measure telomere length has all of these capabilities, which limits their usefulness for practical diagnostic applications.

## DATA AVAILABILITY

The data can be provided by F.X. and W.D.F. pending scientific review, and a completed material transfer agreement. Requests for the data should be submitted to WDF.
